# The social structure of Golfo Dulce bottlenose dolphins (*Tursiops truncatus*) and the influence of behavioural state

**DOI:** 10.1098/rsos.160010

**Published:** 2016-08-31

**Authors:** Kelsey Moreno, Alejandro Acevedo-Gutiérrez

**Affiliations:** 1 Department of Biology, Western Washington University, 516 High Street, Bellingham, WA 98225-9160, USA; 2 Marine Mammal Research Program, Texas A&M University at Galveston, Galveston, TX 77551, USA

**Keywords:** bottlenose dolphins, *Tursiops truncatus*, association patterns, social structure, behaviour, feeding

## Abstract

Ecological factors such as habitat and food availability affect the social structure of bottlenose dolphins (*Tursiops* spp.). Here, we describe the social structure of bottlenose dolphins (*T. truncatus*) in Golfo Dulce, Costa Rica, a semi-enclosed, fjord-like tropical embayment resembling a pelagic system. We also examine behaviour-linked social strategies by comparing social structure relative to behavioural state: feeding versus non-feeding. We analysed 333 sightings over 210 days from boat-based surveys. Despite the uniqueness of the area, the 47 analysed adults had a social structure similar to other populations: a well-differentiated fission–fusion society with sex-specific patterns of associations and aggression. These results indicate that differences in social structure relative to other populations were a matter of degree. Association strength of dyads was highly correlated across behavioural states, indicating constraints on social fluidity. Males displayed a marked difference in lagged association rate and females displayed a small difference in association homogeneity between states. We suggest this difference in population-wide social connections between behavioural states, particularly for males, was due to mating strategies, a pressure which is strongest during non-feeding behaviour and relaxed during feeding. This finding highlights the importance of considering behavioural state when examining individual bonds and the behavioural plasticity for which the bottlenose dolphin is well known.

## 1. Introduction

Social animals exhibit complex networks of social relationships that are composed of associations between individuals [1]. These social relationships are very important because they facilitate transmission of information and disease, and may be crucial for a population's success [1]. Social transmission of information allows for quick adaptation to a dynamic environment [2], such as that found in the ocean. Bottlenose dolphins (genus *Tursiops*) are a great model species to study social networks in animals. They typically reside close to shore and are relatively accessible to study. In part, for this reason a solid body of knowledge about their society already exists (e.g. [3–8]). Bottlenose dolphin society is categorized as fission–fusion, characterized by fluid relationships where individuals associate with a number of other individuals over time [9]. Within this fission–fusion framework, sex-specific patterns are common, and may be due to differences in encounter rate and utilization time of the main reproductively limiting resource of each sex, food for females and mates for males [10]. Typically, males form strong bonds between few individuals while females form loose associations with many individuals [8,10]. Males are more aggressive than females, use aggression in intersexual competition and use sexual coercion during the breeding season [11]. Male harassment is one driving factor for the observed socially or spatially-imposed sexual segregation that occurs in many bottlenose dolphin populations [12]. Other causes may be sex differences in metabolic needs, foraging or learning needs of calves, or protection of calves from predators [12].

Despite the general trends seen in bottlenose dolphin social structure, association patterns vary somewhat between locations, especially for males. In Shark Bay, Australia, males tend to form strong first-order alliances between two and three individuals [10], with second- and third-order alliances forming among primary alliances [6,13]. On the other hand, in Moray Firth, Scotland, the population is dominated by short-term associations, with no evidence of strong alliances [14]. A somewhat intermediate pattern is found in Sarasota Bay, USA, where males use pair-bonding as a normal strategy and non-pair bonding as a transitional strategy [8,15]. By contrast, dolphins in Doubtful Sound, New Zealand, have a uniquely large number of strong, long-lasting, inter- and intrasexual associations, perhaps due to the isolation and highly variable productivity of their fjord habitat [16]. The drivers of variation in social structure between bottlenose dolphin populations are unclear and include resource predictability [9], prey availability [5], resource encounter rates and potential benefits or costs of forming associations [10], rates of immigration and emigration [16] and habitat features [5,16,17]. However, examining the interaction between these different drivers is extremely challenging; one approach is the examination of social structure from the same species in disparate environments [18]. Golfo Dulce is a semi-enclosed tropical embayment located 8–9° N along the Pacific coast of Costa Rica. It is called a tropical fjord due to its deep (greater than 200 m) and subsiding inner basin, which is sheltered from the Pacific Ocean by a shallow (less than 60 m) and stable sill [19]. The embayment is anoxic, with erratic influx of oceanic and fresh waters [20]; the fish fauna is poorly developed and the gulf has low benthic biomass and low overall productivity [21]. Unlike most tropical coastal ecosystems Golfo Dulce is dominated by pelagic biomass and energy flow, resembling an open ocean system rather than an estuarine one [21]. Here we describe the association patterns of bottlenose dolphins in Golfo Dulce. We hypothesized that the unique combination of resource predictability and composition (relatively isolated topography, unpredictable water inflow and pelagic food resources) would also be correlated with a unique social structure.

Studies from dolphin species, including bottlenose dolphins, on variations in association patterns within a population or across behavioural states support the importance of food resources in shaping dolphin sociality. For instance, the different social strategies of Australian snubfin dolphins (*Orcaella heinsohni*) and Indo-Pacific humpback dolphins (*Sousa chinensis*) may be related to differing feeding habitats and prey availability [22]. The use by bottlenose dolphins of anthropogenic food sources such as trawl fishing [23] and fish farming [7] has created social divisions that appear to impact social structure and group cohesiveness. These human-generated food sources result in opportunistic feeding, which reduces levels of association, possibly due to decreased need for cooperation to capture prey [24]. Changes in preferred association between individuals in different behavioural states have been reported in Shark Bay [25]. Males have a high number of preferred associations across all behavioural states and females have few to no preferred associations and a high number of acquaintances [25]. However, the effects of these changes in preferred association on social network structure has yet to be examined. In a different dolphin species, the dusky dolphin (*Lagenorhynchus obscurus*) changes in the group dynamics across behavioural states have also been observed: party size and rate of fusion increased during foraging, while fission rate increased during resting, socializing and travelling [26]. To examine the impact of behaviour-linked social strategies, we describe the social structure of bottlenose dolphins relative to behavioural state.

Our two overall objectives were to characterize the social structure of bottlenose dolphins in Golfo Dulce and to compare it between feeding and non-feeding behavioural states. To accomplish the first objective, we described sex-specific association patterns, community division and lagged association rates and examined sex-specific scarring patterns to reveal levels of aggression. Given the status of Golfo Dulce as a tropical fjord with a distinctive combination of resource predictability and composition, we predicted a unique social structure, similar to the finding of a unique social structure in the fjord habitat of Doubtful Sound [16,27]. To reach the second objective, we determined if social structure differed between feeding and non-feeding behavioural states. As males are expected to be driven by mate acquisition and females by food acquisition, we expected more similarity between behavioural states for males due to mating system constraints and more flexibility between behavioural states for females to maximize foraging success.

## 2. Material and methods

### 2.1. Study site

Golfo Dulce is a semi-enclosed tropical embayment on the southwest coast of Costa Rica categorized as a tropical fjord. The gulf is 50 km long and 15 km wide, covering 750 km^2^ and centred at 8°30′ N and 83°16′ W. It is constituted by a deep inner basin with a maximum depth of 215 m and a shallow outer basin with a sill depth of 60 m, creating a unique environment as one of three basins in the tropics with anoxic conditions and one that resembles a pelagic system rather than a coastal one [19–21]. The embayment supports at least 1028 species [28], including several cetacean species [29,30]. However, only bottlenose dolphins and pan-tropical spotted dolphins (*Stenella attenuata*) reside in the gulf, living sympatrically [31–33]. The two dolphin species are mostly found in different areas of the gulf: bottlenose dolphins tend to frequent shallower waters near shore or rivers, with steep marine slopes, while pan-tropical spotted dolphins tend to occur away from shore in deeper water [31,32]. The distribution of both species varies seasonally, yet no overlap between the two has been observed [31,32]. It is believed that the lack of overlap is due to habitat partitioning as a result of diet discrimination [33]. Although the social structure of bottlenose dolphins from Golfo Dulce has not been previously reported, the first documented case of food-sharing among wild bottlenose dolphins was witnessed in the gulf [34].

### 2.2. Data collection

Our data consisted of 526 bottlenose dolphin sightings (of which 333 were used in analysis) collected over 210 days between September 1991 and December 1992 from boat-based surveys as described in Acevedo-Gutiérrez & Burkhart [31]. The non-random boat surveys were conducted on board two inflatable boats (less than 5 m long) each powered by a 25 hp outboard engine. Surveys were conducted an average of 5 days per week and effort was made to cover the entire study area each week. The methodology employed to observe dolphins is described in Acevedo-Gutiérrez [35]. Each group of dolphins sighted was considered a focal group and followed for as long as possible while identifying individual dolphins through photographs of both sides of their dorsal fins [36] and recording location, size of group and behavioural state. Group-follows lasted 94.67 ± 73.69 min and ended ad libitum [37] when dolphins were lost or weather conditions prevented data collection. A group of dolphins was defined based on the 10-m chain rule [3], any dolphin <10 m (about two vessel lengths) of any other dolphin was considered part of the same group. Group membership was continuously recorded. Dolphin groups rarely split during our follows, when it happened we followed the largest remaining group.

The description of behavioural states is presented in Acevedo-Gutiérrez [35] and Acevedo-Gutiérrez & Parker [38]. Briefly, we scan-sampled six previously defined behavioural events (size of subgroups, orientation, speed, diving, synchrony of diving and aerial behaviour) constantly when dolphins were at the surface. This sampling was possible because the median size of focal groups was less than six dolphins. We avoided re-sampling individuals within a surfacing period by keeping track of their positions while at the surface. The combined data from all six behavioural events in a surfacing period was termed a surfacing bout. Each bout defined a behavioural state that lasted for as long as consecutive surfacing bouts represented that state. If a group switched behavioural states, we used the duration of each state to determine the predominant group activity performed by the majority of the individuals [39]. We defined feeding as the majority of dolphins visibly pursuing fish or holding fish in their mouths. Non-feeding activities included travelling, socializing, and milling. During travelling, dolphins consistently swam in a general direction, with rhythmic surfacing cycles and without extended dives. During socializing, dolphins moved irregularly, usually remaining in one general location, had irregular surfacing cycles without extended dives, frequently leaped and splashed against the surface of the water, and rubbed and touched one another. During milling, dolphins moved irregularly and slowly in one general location, with rhythmic surfacing cycles and without extended dives and with no visible interaction with one another. We view as improbable that we missed dolphin feeding episodes given the large frequency of feeding episodes that we recorded and the clear, distinctive nature of the other behavioural states. Each sighting was tallied as feeding or non-feeding if the group was engaged in that activity for greater than 50% of the time [40]. In the rare cases in which feeding and non-feeding activities had the same duration, the sighting was excluded from analysis.

In the laboratory, we tallied for each sighting the individual observed and the percentage of group members identified. Photographs of the dorsal fin were taken on B/W film or colour slides with a Canon T90 SLR camera and either a FD 80–200 mm *f*/4 or a FD 100–300 mm *f*/5.6 Canon zoom lens. To identify individuals, we only employed focused and well-exposed photographs parallel or almost parallel to the plane of the camera. Afterwards we selected images with distinctive nicks, notches or scars on the fin for identification purposes. As a result, images of distinctive fins were employed even if they were not completely parallel to the plane of the camera, whereas perfectly parallel shots of undistinctive images were not selected for analysis. The sex and age of each individual was tallied as male, female or unknown, and adult, young, calf or neonate. Males were categorized based on the presence of a visible penis; females based on continuous and close association throughout the 16-month study period with a young, calf or after birth, a neonate. Age was categorized based on relative body length, with calves having less than 1/3 and young between 1/3 and 2/3 the length of an adult; neonates were defined as calves showing fetal folds.

### 2.3. Overall social structure

#### 2.3.1. Association patterns

We followed the general methodology employed in studies of the social structure of dolphins [22,41–43]. Individuals were considered associated on any survey day if they were photographed within the same focal group on that day (the sampling interval). Only the first sighting per sampling period was included in the analysis. We only included in the analysis adult individuals sighted ten or more times to reduce data skew from individuals rarely sighted, thus enhancing the likelihood of accurately describing their social structure [44]. Immature individuals (young, calves and neonates) were excluded from the analysis as they were both primarily associated with one adult and did not provide additional information regarding the overall social structure. In certain cases, we were unable to identify all individuals in a group. To avoid underestimating association patterns, we only included in the analysis groups for which we were able to identify, based on the photographs taken, greater than or equal to 50% of individuals at the time of the sighting [22].

We analysed the data to describe overall social structure, including analyses of associations relative to sex, and to compare it between behavioural states with Socprog 2.4 [45]. The most basic components of a social network are dyadic associations, which can elucidate social strategies of individuals and their influence on population-level dynamics [1]. Dyadic associations are therefore the centre of most network research and thus the focus of our study. Because social networks are intrinsically weighted, we used weighted network analysis to better understand the social structure of Golfo Dulce bottlenose dolphins [22,46,47]. We weighted the links between individuals based on association strength, which was measured with the half-weight index (HWI). We chose this index to provide a symmetrical association measure with reduced bias given that under the sampling techniques employed pairs would have a greater likelihood of being scored when separate than when together [48]. Values of the index range from 0 to 1, where a value of 0 indicates that the individuals were never observed together and a value of 1 indicates that they were always observed together.

The proportion of time that dyads actually spend together may be quite different from the association indexes estimated from observational data [22]. Thus, the matrices calculated from surveys may not be accurate representations of the social structure. To assess the accuracy of the data in describing the social structure, we used Socprog to calculate the estimate of correlation between true (proportion of time dyads actually spend together) and estimated (sampled) association indexes using a likelihood approximation and bootstrapped standard error [22,45,49]. The correlation shows the power of the analysis to describe the social system: values close to 1 indicate a very good representation and values near 0.4 indicate a moderate representation [45]. The estimate of the correlation between true and estimated association indices was 0.790 (s.e. = 0.019), indicating that the data accurately represented the association patterns of the population.

We were thus able to describe the weighted association network using several analyses. To determine the level of social differentiation in the population, a coefficient of variation was calculated in Socprog using the formulas and likelihood methods described in Whitehead [45,49]. Values <0.3 indicate low levels of social differentiation while values >0.5 indicate high levels of social differentiation [45]. In addition, two network metrics were calculated: strength and clustering. Strength is a metric indicative of an individual's tendency to form associations [22]. It is calculated by summing the association indexes of an individual with each of their associates [22]. Clustering is a measure of how strongly associated an individual's associates are among themselves. Following Parra *et al*. [22], we calculated it using Holme's formula:

$$C_{i}=\frac{\sum_{jk}AI_{ij}AI_{ik}AI_{jk}}{\max(AI_{ij})\sum_{jk}AI_{ij}AI_{jk}}.$$

To determine the significance of these network metrics as well as the standard deviation, mean and non-zero proportion of the association indices (HWI) of the population, we ran 20 000 permutations with 1000 trials per permutation to generate a null population with which we compared values calculated from the true population. Values from the population were considered significantly different from the null population if greater than or equal to 95% of generated values were either higher or lower than the measured values. High population mean values for HWI indicate the population has stronger associations on average than would be expected if all association were random. A high population standard deviation for HWI indicates a greater spread in association values than would be expected if all associations were random, and thus indicate the existence of preferential associations. Low mean values indicate weaker associations on average than random, and low population standard deviations indicate associations are more similar in strength between individuals than would be expected if all associations were random, and thus indicates the existence of equal preference or no preference for potential associates. The proportion of non-zero elements captures how many of the possible associations are present. A low proportion of non-zero elements indicates fewer associations than would be expected if all associations were random, and thus indicates the presence of individuals that avoid associating with one another. High values indicate the presence of individuals that do not avoid associating with one another. The permutation test was conducted in Socprog 2.4 based on Bejder *et al*. [44]. The method employed by Socprog 2.4 uses an algorithm that swaps values in a manner ensuring that the generated matrices keep certain features constant, such as the number of individuals in a group and the number of sightings for each individual [44]. Following Parra *et al*. [22], we permuted individuals among groups within each sampling period to remove the effects of lack of independence in group membership. Permutations were also employed for associations within and between the sexes. Additionally, a Mantel test was used to determine whether association rates between and within sex classes were similar [45]. To visualize overall patterns in the population and associations between individuals, we plotted three sociograms using Netdraw [50]: one for the population, one for males and one for females. Paired males were defined as reciprocal closest associate with an HWI above 0.5 following Owen *et al.* [15].

#### 2.3.2. Community division

We divided the population into groups using community division by modularity, which can show social units within the population [22]. This technique is based on calculating the edges that connect different communities, removing them and repeating the procedure to give a succession of splits of the entire network [47]. The number of communities yielded is based on which division has the best modularity coefficient, thereby maximizing association values within members of the same community and minimizing association values between members of different communities [47]. A coefficient ≥0.3 is considered indicative of a beneficial division [45]. The modularity coefficient is calculated using the fraction of edges within the community minus the expected value if the network was random [47]. The coefficient is given by the formula:

$$Q=\frac{\sum_{ij}\propto_{ij}\delta (C_{i},C_{j})}{\sum_{ij}\propto_{ij}}-\frac{\sum_{ij}{\hat{\propto }}_{ij}\delta (C_{i},C_{j})}{\sum_{ij}{\hat{\propto }}_{ij}},$$

where $\propto_{ij}$ is the index of association between individuals *i* and *j*, and $\propto_{ij}$ is the expected association index between the same two individuals if random association is occurring [22]. Additionally, $\delta (C_{i},C_{j})$ is either 1 if *i* and *j* are members of the same cluster or 0 if they are not [22].

#### 2.3.3. Lagged association rates

We calculated the standardized lagged association rate (sLAR) to determine the temporal association patterns of the population. The sLAR is an estimate of the probability that if two individuals are associated at any time, the second animal is still associated with the first after the specified lag [22,45,49]. A null sLAR showing the expected sLAR given random associations was calculated for comparison with the sLAR of the population. Best-fit models for sLARs were calculated using a number of previously derived model frameworks used to describe social structures [45,49]. The model of best fit was selected using the quasi-Akaike information criterion (QAIC); the model with the lowest QAIC and any models with a value < 2 above the lowest QAIC value were considered to have sufficient support.

#### 2.3.4. Scarring

Determining the relative levels of aggression towards individuals in a population is particularly useful in determining the presence of male–male competition or female coercion; variations in these factors are linked to variations in bottlenose dolphin mating strategies and hence their social behaviour [51]. Intraspecific scarring is a useful indicator of aggression towards an individual [11]. We measured the long, parallel scars known as tooth-rakes, which are characteristic of dolphin bites and indicative of intraspecific aggression [52]. Specifically, they are indicative of relatively recent aggressions, as in bottlenose dolphins scars re-pigment less than 2 years from infliction and, therefore, do not accumulate over an animal's lifetime [52].

To quantitatively measure the scarring on each individual, we transformed the original photos into digital images. We only employed photos of adult individuals of known sex; for consistency, we selected the best photograph from the dolphin's left side: perpendicular to the lens, properly illuminated, well focused and completely showing the dorsal fin. We only employed a single photograph for each identified individual, selecting the best-quality image based on light exposure and camera angle. The selection of the single photograph employed was independent of the amount of scarring on the fin. Using Adobe Photoshop CS6, we coloured all the area occupied by tooth-rakes and then traced the silhouette of the dorsal fin, filling in notches and following the contour line of the body at the base of the fin. We decided to estimate the amount of scarring relative to the surface area of a complete dorsal fin to standardize all different fins. We recognize that this conservative approach reduces the percentage of scarring in some cases, but it eliminates the possibility of a small scar yielding a large percentage of scarring because part of the fin is missing. Giving that we were unable to determine the reason(s) for the nicks, scars and portions of a fin missing, we believe that the conservative approach we employed fairly reflects the amount of scarring within the study population. We then compared the number of pixels occupied by the raking pattern on dorsal fins with those occupied by the total fin area to obtain a relative measure of scarring. As the data were non-normal, we transformed them with an arcsine transformation, then used ANOVA to compare the percentage of scarring between males, females with a neonate and females without a neonate (but with a calf or a young). In a posterior analysis, we ran planned comparisons (contrasts) of the amount of scarring between females and males, and between females with a neonate and females without a neonate.

### 2.4. Feeding and non-feeding social structure

To determine if association patterns differed between feeding and non-feeding behavioural states, the sighting data described under data collection were separated by behavioural state of the group and the HWI matrix was generated for each behavioural state. A *t*-test confirmed that the ratio of males to females per sighting was similar between behavioural states. Using the newly generated indices, we tested for correlation of the HWI for all dyads between behavioural states with a Mantel test [45]. We also performed the same tests described for the overall structure to depict the social structure exhibited during each behavioural state and to compare it between states. Comparison between states for male–male associations and female–female associations were used to test hypotheses on how sex-specific primary resources shape social structure while population-level comparisons and intersexual association comparisons were exploratory. All analyses were performed in Socprog 2.4 for Matlab [45]. Additionally, group sizes between behavioural states were compared in R v. 2.15.1 [53] using a Mann–Whitney *U* test given that the data did not follow a normal distribution.

## 3. Results

### 3.1. Overall social structure

**Figure 1.**
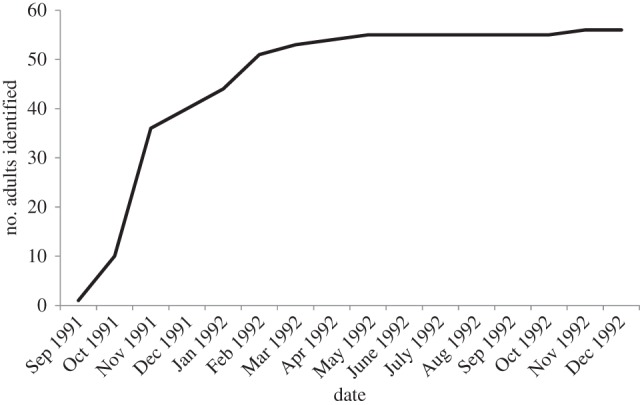
Number of new individuals identified throughout the study period.

Out of 526 total sightings, we included in all analyses 333 sightings of groups with greater than or equal to 50% of individuals identified. Group size ranged from 1 to 25 individuals with an average of 6.9 ± s.d. 4.5 individuals (*n* = 334 groups). We classified 25 adult females, 11 adult males, 20 adults of unknown sex, 9 young, 3 calves and 11 neonates (all born between April and Sep 1992). Of 56 adults identified, we only included 47 adults observed greater than or equal to 10 times: 23 females, 11 males and 13 adults of unknown sex. Close to 64% of adults had been identified within the first two months of the field study and 91% of the individuals were identified well before the half-point mark of the study (figure 1).

#### 3.1.1. Association patterns

The society was well differentiated, as shown by the estimate of social differentiation, which measured 0.915 (s.e. = 0.028). Calculated network metrics had a significantly higher strength coefficient than a random population (Golfo Dulce = 4.96, random = 4.95, *p* < 0.001), indicating marginally stronger than random associations among individuals, but there was no significant difference for the clustering coefficient (Golfo Dulce = 0.23, random = 0.22, *p* = 0.35), indicating that the associates of an individual were not associated with one another more than would be expected by chance. Permutation tests indicated that dolphins preferentially associated with some members of the population and avoided others: the standard deviation was significantly higher than would be expected by random (table 1). The mean HWI for the population was also higher than random (table 1), further demonstrating strong associations. The proportion of non-zero elements was significantly smaller than random (table 1), indicating avoidance between individuals.

**Table 1.** The mean, standard deviation and proportion of non-zero elements for the measured population HWI values and the average of the mean, standard deviation and proportion of non-zero elements for the HWI values generated by each of 20 000 permutation runs. The *p*-values are based on the proportion of permutation runs above or below the population value. Population values significantly different from generated values are indicated with an asterisk.

	population	random POP	*p*-value	male	random male	*p*-value	female	random female	*p*-value	male– female	random M–F	*p*-value
mean	0.10773*	0.10762	0.005	0.21678	0.21690	0.171	0.10596	0.10600	0.290	0.10556	0.10558	0.211
s.d.	0.12480*	0.11141	<0.001	0.17986*	0.17297	<0.001	0.11560*	0.10439	<0.001	0.10404	0.10337	0.880
non-zero	0.75763*	0.81534	<0.001	0.98182	0.98691	0.360	0.75099*	0.81378	<0.001	0.84980	0.85220	0.364

**Figure 2.**
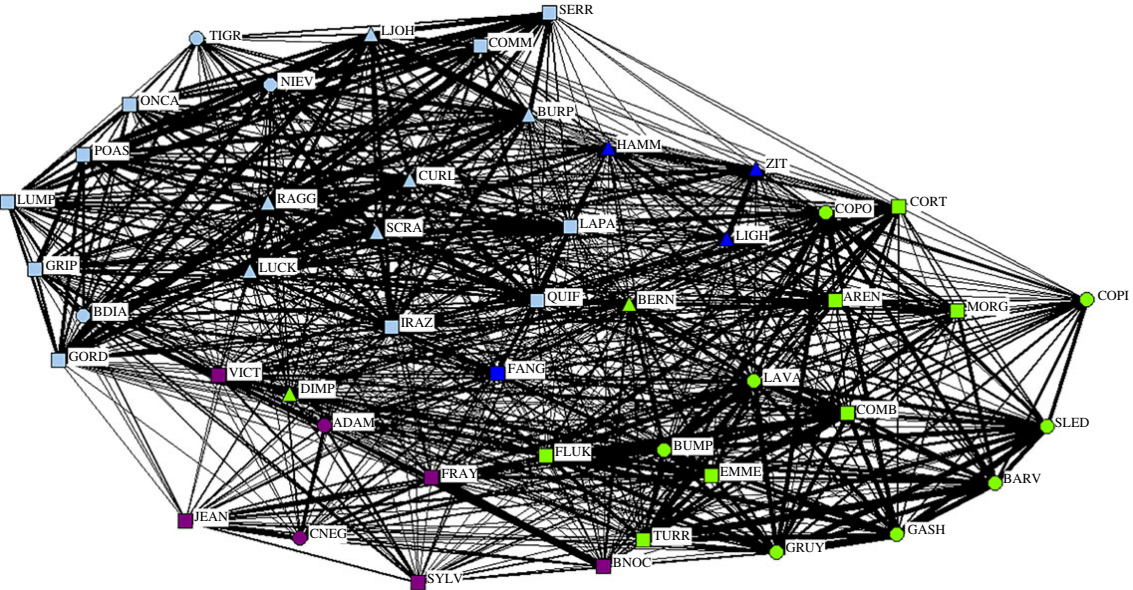
Sociogram displaying dyadic associations between identified Golfo Dulce bottlenose dolphins. Individuals are labelled with a four letter name. Individuals are coloured by cluster as determined by cluster analysis by modularity. Sex is indicated by shape: males are triangles, females are squares and adults of unknown sex are circles. The thickness of the line between individuals indicates the level of the strength of the association.

We detected sex-specific association patterns among bottlenose dolphins in Golfo Dulce. Mean HWIs for female–female, male–male or male–female associations were not significantly different from random (table 1). However, the average HWI was higher for male–male dyads than for female–female or male–female dyads. The standard deviation of the HWIs was significantly greater than random for both male–male and female–female associations, but not for male–female associations (table 1), indicating preferred intrasexual rather than intersexual associations. The proportion of non-zero elements was significantly smaller than random for female–female associations but not for male–male or male–female associations (table 1), indicating that avoidance was only a feature of female–female associations. The sociogram of the overall population structure allowed us to visualize the associations between individuals, and indicated a well-connected population with varying strengths of associations, graphically supporting the results found above (figure 2).

**Figure 3.**
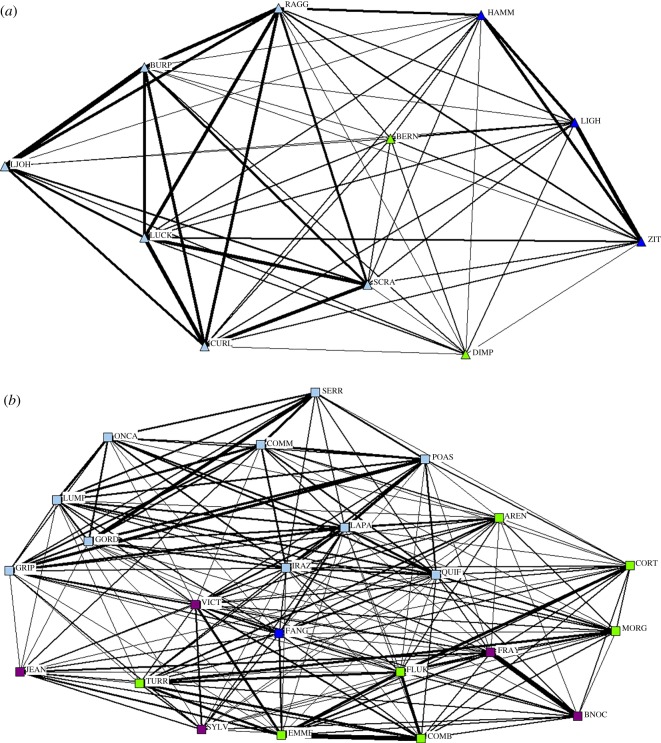
(*a*,*b*) Sociograms by sex. Males are displayed above as triangles and females are displayed below as squares. Individuals are coloured by cluster and labelled with a four letter name. The thickness of the line between individuals indicates the level of the strength of the association.

Average association values within sex were higher than those between sexes (Mantel test: within = 0.15, between = 0.09, *p* < 0.001). The sociograms by sex indicate that males appeared to form few but strong associations with one another while females appeared to form many but loose associations with one another (figure 3). There were three sets of paired males: LOJH & BURP, LUCK & CURL and LIGH & ZIT. However, all paired males also had strong associations with at least one other male, thus pairs were not isolated units, but formed the basis of two main interconnected groups (figure 3). Additionally, the two males without a single strong association still had multiple weak connections to both groups.

#### 3.1.2. Community division

A cluster analysis through community modularity divided the population into four groups of varying sizes and sex compositions (figure 2). A modularity of 0.333 indicates that the clusters were a useful division of the population.

#### 3.1.3. Standardized lagged association rates

**Figure 4.**
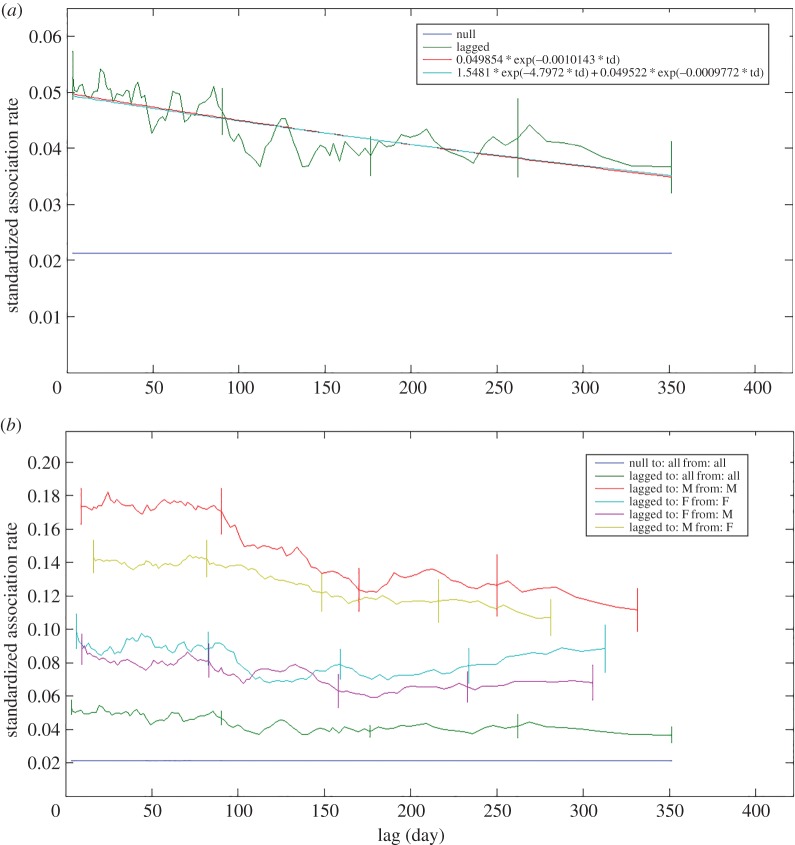
Standardized lagged association rate (sLAR) relative to time in days of identified Golfo Dulce bottlenose dolphins. Vertical lines show margin of error through jackknifing. Included for comparison is the null sLAR. sLAR is shown for (*a*) all adults with the best-fit model with parameters displayed in the inset, as well as for (*b*) each sex combination.

Temporal association patterns showed various levels of associations (figure 4). Two models were determined to have sufficient support using the set selection criteria and the quasi-AIC. These were the two levels of casual acquaintances model, and the casual acquaintances model (table 2) indicating that long-term, unchanging associations probably did not play a significant role in population temporal patterns. Male–male associations were fitted to the casual acquaintances model, with some support for the constant companions and casual acquaintances model (table 2). Only the constant companions and casual acquaintances model was supported for female–female associations (table 2). Male–female associations were best fitted by the casual acquaintances model, with some support for the two levels of casual acquaintances model (table 2), while only the casual acquaintances model was supported for female–male associations (table 2).

**Table 2.** Lagged association rate fit models for the full population and for associations between and within sexes. Fit models have their equation provided and are described using a name which corresponds to the type of associations which may shape the equation in the observed way. QAIC and ΔQAIC are used to determine model fit. Supported models are indicated in bold italics.

	name	model	QAIC	ΔQAIC
all	constant companions	0.044427	64034.8022	64.2948
	***casual acquaintances***	0.049854*exp(−0.0010143*td)	***63971***.***4037***	***0***.***8963***
	constant companions + casual acquaintances	0.044212 + 0.061497*exp(−1.2112*td)	64027.1393	56.6319
	***two levels of casual acquaintances***	1.5481*exp(−4.7972*td) + 0.049522*exp(−0.0009772*td)	***63970***.***5074***	***0***
male–male	constant companions	0.15149	36767.6784	176.9371
	***casual acquaintances***	***36590.7413***	***36590***.***7413***	***0***
	***constant companions*** + ***casual acquaintances***	0.013848 + 0.17081*exp(−0.0018233*td)	***36592***.***723***	***1***.***9817***
	two levels of casual acquaintances	−0.028558*exp(−0.0016191*td) + 0.21283*exp(−0.0016409*td)	36594.7413	4
female–female	constant companions	0.083301	19959.526	12.9452
	casual acquaintances	0.088944*exp(−0.00055752*td)	19953.9516	7.3708
	***constant companions*** + ***casual acquaintances***	0.077008 + 0.022351*exp(−0.017003*td)	***19946***.***5808***	***0***
	two levels of casual acquaintances	0.057094*exp(−0.66933*td) + 0.087708*exp(−0.00048111*td)	19954.8757	8.2949
male–female	constant companions	0.07417	15660.9338	18.3211
	***casual acquaintances***	0.08396*exp(−0.0010458*td)	***15642***.***6127***	***0***
	constant companions + casual acquaintances	0.07429 + (−447.6758)*exp(−10.1249*td)	15663.8653	21.2526
	***two levels of casual acquaintances***	−46.4202*exp(−7.4096*td) + 0.084618*exp(−0.001089*td)	***15644***.***2228***	***1***.***6101***
female–male	constant companions	0.129	17574.6855	39.089
	***casual acquaintances***	0.14913*exp(−0.0012124*td)	***17535***.***5965***	***0***
	constant companions + casual acquaintances	0.12868 + 558.4714*exp(−8.5328*td)	17572.7035	37.107
	two levels of casual acquaintances	0.1571*exp(−1.3236*td) + 0.14831*exp(−0.0011822*td)	17538.1862	2.5897

#### 3.1.4. Scarring

The amount of scarring on the dorsal fins differed among sex classes (ANOVA_2,23_ = 7.92, *p* = 0.003). Males had the greatest scarring (10.2 ± s.d. 1.17%), followed by females with neonates (2.2 ± s.d. 2.78%); the least scarring occurred on females with a calf or a young (0.42 ± s.d. 1.36%). There was a significant difference in the amount of scarring between males and females (contrasts: *t*_20_ = 4.10, *p* < 0.001), but not between females with a neonate and females with a calf or a young (contrasts: *t*_20_ = 1.29, *p* > 0.05).

### 3.2. Feeding and non-feeding social structure

**Figure 5.**
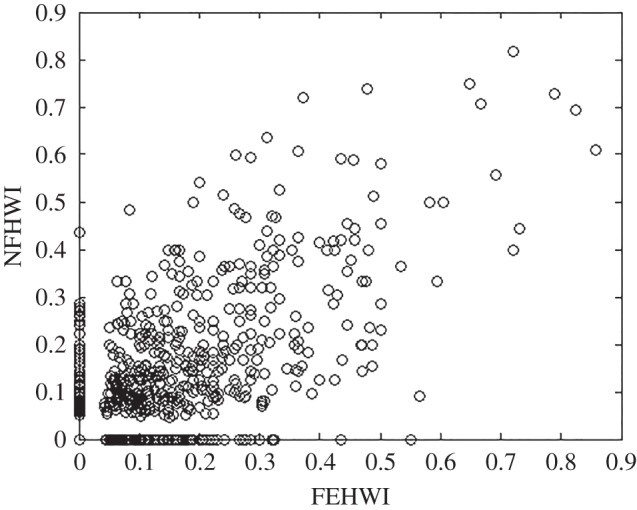
Correlation between feeding (FEHWI) and non-feeding (NFHWI) half-weight index for all dyads of identified Golfo Dulce bottlenose dolphins. Each circle represents a dyad.

Sighting data were divided between 177 observations of animals engaging in feeding behaviours and 153 observations of animals engaging in non-feeding behaviours. Social structure between feeding and non-feeding behavioural states had high degrees of similarity and few differences. The HWI of dyads were strongly correlated between behavioural states (Mantel *Z*-test: value of the correlation = 1, *n* = 20 000 permutations, *p* < 0.001; figure 5). Group size was not significantly different between behavioural states (Mann–Whitney test: feeding = 6.8 ± s.d. 4.2 individuals, *n* = 177 groups; non-feeding = 7.0 ± s.d. 4.8 individuals, *n* = 153 groups; *W *= 3635.5, *p* = 0.9122). The standard deviation of both behavioural states was significantly higher than random (table 3), indicating preferred associations during both behavioural states. The proportion of non-zero elements was significantly lower than random for both behavioural states (table 3), indicating avoidance between individuals during both behavioural states.

**Table 3.** The mean, standard deviation and proportion of non-zero elements for the measured population HWI values and for the HWI values generated by each of 20 000 permutation runs. Population values and random values are separated by behaviour. The *p*-values are based on the proportion of permutation runs above or below the population value. Population values significantly different from generated values are indicated with an asterisk. Values which are significant in one behavioural state and not the other are indicated in bold.

		random			random			random		male–	random	
	population	POP	*p*-value	male	male	*p*-value	female	female	*p*-value	female	M–F	*p*-value
feeding												
mean	0.11032	0.11032	0.515	0.20435	0.20456	0.075	0.10721	0.10720	0.543	0.10721	0.10722	0.286
s.d.	0.13326*	0.12685	<0.001	0.17233*	0.16703	0.003	0.12646*	0.12040	<0.001	0.10371	0.10228	0.137
non-zero	0.63922*	0.66357	<0.001	0.85455	0.85455	0.502	**0**.61660*	0.64933	<0.001	0.75099	0.75541	0.247
non-feeding												
mean	**0**.09746*	0.9711	<0.001	**0**.20372*	0.20317	0.029	0.09700	0.09701	0.497	**0**.09672*	0.09649	0.006
s.d.	0.13563*	0.12734	<0.001	0.21758*	0.21101	0.002	0.12681*	0.12250	0.010	0.12719	0.12652	0.065
non-zero	0.51434*	0.54845	<0.001	0.78182	0.80778	0.060	0.54150	0.54631	0.220	0.52174	0.52623	0.087

Most sex-specific associations were also similar between behavioural states. The mean HWI for female–female association was not significantly different from random for either state (table 3). The standard deviation for same-sex association was significantly higher than random in both behavioural states (table 3). The standard deviation for mixed-sex association was not significantly different from random for both behavioural states (table 3). Sex proportions were similar between the behavioural states (*t*-test *p* = 0.253), hence observed differences were not due to differences in sample size.

**Figure 6.**
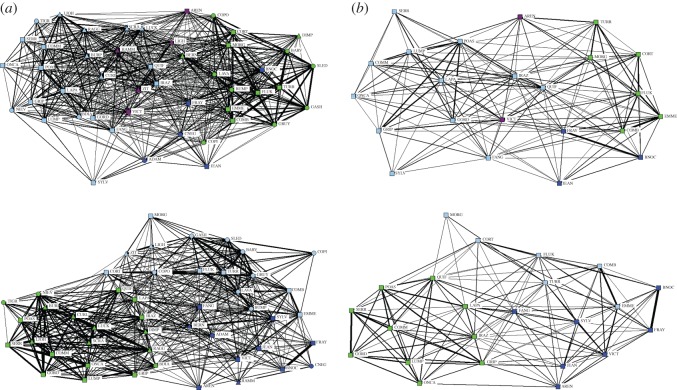
Sociograms of identified Golfo Dulce bottlenose dolphins relative to behavioural state. Feeding above, non-feeding below. Individuals are shown as squares and labelled with a four letter name. The thickness of the line between individuals indicates the level of the strength of the association. (*a*) All individuals are included and coloured by cluster. Sex is indicated by shape: males are triangles, females are squares and adults of unknown sex are circles. (*b*) Sociogram of female–female interactions.

There were a couple of small differences between the behavioural states in the division of the community. The sociograms show slight differences in individual connections (figure 6). The feeding sociogram shows more individuals with connections than the non-feeding sociogram while the non-feeding sociogram displays two clear clusters of strong associations; as result the former has a busy look whereas the latter has a clumped one (figure 6*a*). However, these slight differences had a minimal impact on the overall structure, as both sociograms still exhibit key overall features of a well-connected population and variation in associations (figure 6*a*). Sociogram structure differences became more pronounced when the analysis focused on female–female associations (figure 6*b*). During non-feeding these associations split into two main subgroups with weak connections between them, whereas during feeding the associations indicated a single, strongly connected group (figure 6*b*). Cluster analysis shows slight changes in group composition and fewer clusters during non-feeding behaviour than during feeding (figure 6).

Regarding the overall population, small differences also occurred in the HWI of the associations. During non-feeding, the HWI of the population-wide associations was higher than random (table 3). However, that was not the case when dolphins were feeding (table 3). There was also a difference in the mean HWI for male–male and male–female associations. For both, the mean HWI was higher than random during non-feeding (table 3), but not during feeding (table 3). Additionally, only female–female association displayed a difference in the proportion of non-zero elements between behavioural states, with lower than random proportions of non-zero elements during feeding, but not during non-feeding behaviour (table 3).

**Figure 7.**
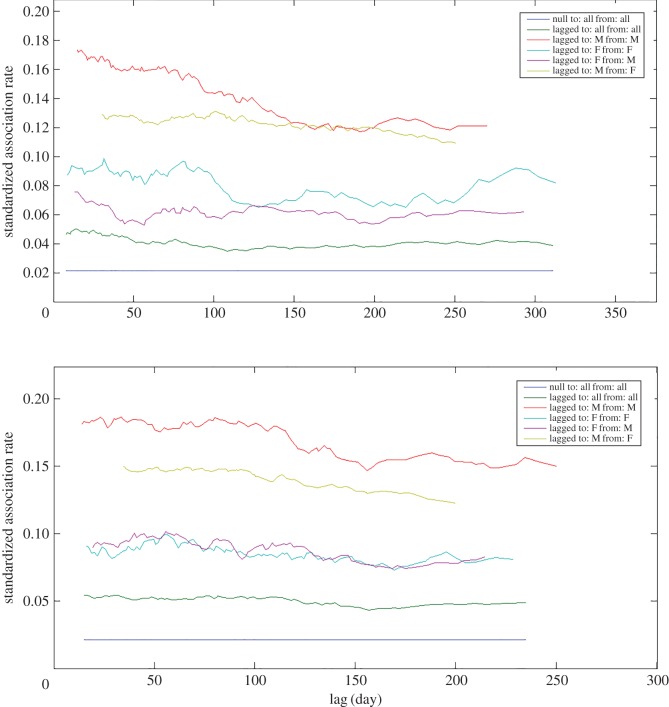
Standardized lagged association rate (sLAR) of identified Golfo Dulce bottlenose dolphins relative to behavioural state. Feeding above, non-feeding below. The sexes that were associated with one another are displayed in the inset. Included for comparison is the null sLAR.

**Figure 8.**
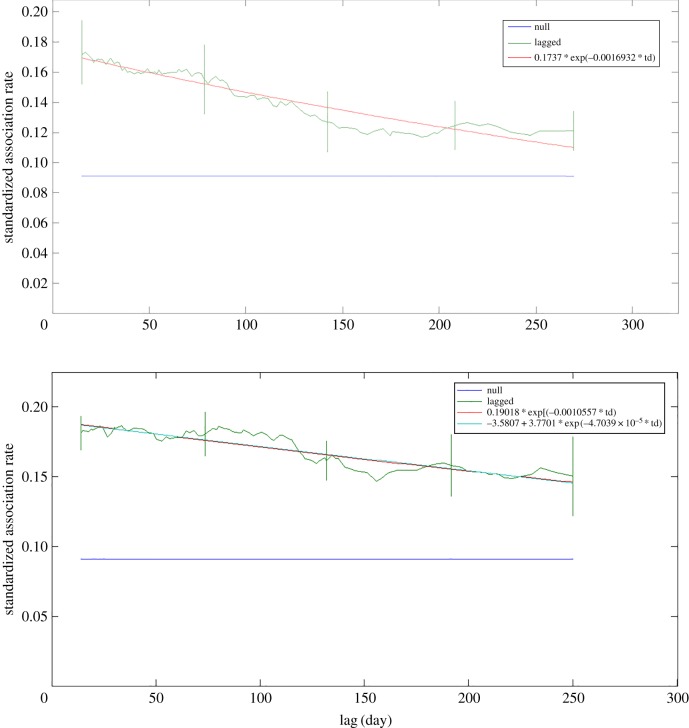
Standardized lagged association rate (sLAR) of identified Golfo Dulce bottlenose dolphin males relative to behavioural state. Feeding above, non-feeding below. The parameters of the best-fit models are displayed in the inset. Included for comparison is the null sLAR. Vertical lines show margin of error through jackknifing.

Differences between behavioural states were found in the sLAR between sex combinations, particularly for male–male associations (figures 7 and 8). During non-feeding behaviour, males displayed a markedly higher and more stable lagged association rate (figures 7 and 8). However, during feeding, the males were more likely to disassociate sooner, as illustrated by the steepness of the curve (figure 8). The best-fit models for each lagged association rate also differed. During feeding, the male association data were only substantially supported by the casual acquaintances model (table 4). The best fit for the data during non-feeding behaviour was provided by a model described as constant companions and casual acquaintances, though there was also support for the casual acquaintances model (table 5). The parameters of the model of casual acquaintances during feeding were different than during non-feeding, resulting in a greater decay in association rate in the former than the latter. The initial association rate during non-feeding was also higher than during feeding.

**Table 4.** Lagged association rate fit models for the full population and for associations between and within sexes during feeding behaviour. Fit models have their equation provided and are described using a name which corresponds to the type of associations which may shape the equation in the observed way. QAIC and ΔQAIC are used to determine model fit. Supported models are indicated in bold italics.

feeding	name	model	QAIC	ΔQAIC
all	constant companions	0.041284	20777.5	10.5515
	***casual acquaintances***	0.044794*exp(−0.0007261*td)	***20767***	***0***
	constant companions + casual acquaintances	0.041022 + 0.059773*exp(−1.2065*td)	20776.3	9.305
	***Two levels of casual acquaintances***	0.13925*exp(−2.1963*td) + 0.044331*exp(−0.00067171*td)	***20767***.***5***	***0***.***4955***
male–male	constant companions	8128.1889	8161.06	36.8673
	***casual acquaintances***	0.1737*exp(−0.0016932*td)	***8124***.***19***	***0***
	constant companions + casual acquaintances	0.14335 + 0.21202*exp(−1.3256*td)	8162.97	38.777
	two levels of casual acquaintances	−0.056187*exp(−0.0016945*td) + 0.2299*exp(−0.0016941*td)	8128.19	4
female–female	***constant companions***	***0.082764***	***6314***.***74***	***0***.***829***
	***casual acquaintances***	0.088511*exp(−0.00055287*td)	***6313***.***91***	***0***
	constant companions + casual acquaintances	0.082361 + 546.2604*exp(−9.4193*td)	6316.63	2.7152
	two levels of casual acquaintances	0.01872*exp(−0.22725*td) + 0.086966*exp(−0.00046399*td)	6317.36	3.4452
male–female	***constant companions***	***0.062842***	***5410***.***85***	***0***
	***casual acquaintances***	0.066531*exp(−0.00046539*td)	***5411***.***25***	***0***.***4038***
	constant companions + casual acquaintances	0.062559 + 0.10749*exp(−1.2188*td)	5413.96	3.1094
	two levels of casual acquaintances	0.944*exp(−4.0096*td) + 0.066315*exp(−0.00044935*td)	5415.01	4.1623
female–male	constant companions	0.12314	4390.08	3.3881
	***casual acquaintances***	0.1362*exp(−0.00080685*td)	***4386***.***69***	***0***
	constant companions + casual acquaintances	0.1226 + 4194.7413*exp(−10.0039*td)	4390.1	3.4115
	two levels of casual acquaintances	0.070015*exp(−0.73399*td) + 0.13505*exp(−0.00076325*td)	4390.3	3.6062

**Table 5.** Lagged association rate fit models for the full population and for associations between and within sexes during non-feeding behaviour. Fit models have their equation provided and are described using a name which corresponds to the type of associations which may shape the equation in the observed way. QAIC and ΔQAIC are used to determine model fit. Supported models are indicated in bold italics.

non-feeding	name	model	QAIC	ΔQAIC
all	constant companions	18984.9495	18984.9	2.4339
	***casual acquaintances***	0.053783*exp(−0.00054627*td)	***18982***.***5***	***0***
	constant companions + casual acquaintances	0.050626 + 0.055868*exp(−1.2156*td)	18987.1	4.6072
	two levels of casual acquaintances	0.27114*exp(−3.3407*td) + 0.053557*exp(−0.00052082*td)	18985.9	3.4315
male–male	constant companions	0.16953	10129.2	15.4575
	***casual acquaintances***	0.19018*exp(−0.0010557*td)	***10114***.***1***	***0***.***423***
	***constant companions*** + ***casual acquaintances***	−3.5807 + 3.7701*exp(−4.7039×10^-5^*td)	***10113***.***7***	***0***
	two levels of casual acquaintances	0.014385*exp(−1.1935*td) + 0.19004*exp(−0.0010503*td)	10116.1	2.4135
female–female	***constant companions***	***0.086816***	***3515***.***87***	***0***
	***casual acquaintances***	0.090594*exp(−0.00041328*td)	***3517***.***3***	***1***.***4376***
	***constant companions*** + ***casual acquaintances***	0.085251 + 0.046123*exp(−0.18351*td)	***3517***.***55***	***1***.***6887***
	two levels of casual acquaintances	0.36642*exp(−2.3863*td) + 0.08956*exp(−0.00034102*td)	3520.62	4.753
male–female	***constant companions***	***0.089062***	***3172***.***34***	***0***
	***casual acquaintances***	0.092137*exp(−0.00032373*td)	***3174***.***03***	***1***.***695***
	constant companions + casual acquaintances	0.089291 + (−419.9642)*exp(−9.8295*td)	3175.96	3.6243
	two levels of casual acquaintances	−26.4383*exp(−6.9198*td) + 0.092993*exp(−0.00038142*td)	3177.55	5.2119
female–male	constant companions	0.13848	4439.54	6.4011
	***casual acquaintances***	0.15803*exp(−0.0012177*td)	***4433***.***14***	***0***
	constant companions + casual acquaintances	0.13657 + 0.022453*exp(−0.018025*td)	4440.58	7.4392
	two levels of casual acquaintances	0.034556*exp(−0.97932*td) + 0.15748*exp(−0.0011957*td)	4437.08	3.9465

Temporal associations also differed between behavioural states for male–female associations, although which sex was analysed as the to or from group resulted in greater differences than behavioural state. The association rate during non-feeding behaviour was higher and more constant over time than during feeding for both male to female associations and female to male associations (figure 7). However, associations from males to females were consistently more likely and more stable over time than associations from females to males (figure 7). Temporal associations during feeding from males to females were best fitted by the constant companions model, with less support for the casual acquaintances model (table 4). Temporal associations during non-feeding behaviour from males to females were only fitted by the constant companions model (table 5). Temporal associations from females to males were best fitted by the model of casual acquaintances during both feeding (table 4) and non-feeding (table 5) behaviour.

The sLARs also differed slightly between behavioural states for female–female associations. Although association probability over time was very similar between feeding and non-feeding behaviours (figure 7), the fit models demonstrated that the change over time is slightly different. Temporal patterns during feeding had the most support for the casual acquaintances model and less support for the constant companions model (table 4), while temporal patterns during non-feeding only supported the constant companions model (table 5).

## 4. Discussion

### 4.1. Overall social structure

Our results indicate that bottlenose dolphins in Golfo Dulce were organized into a well-differentiated fission–fusion society, with strong evidence of assortative mixing by sex as indicated by the higher within-class than between-class association values. This pattern is common among bottlenose dolphin populations [3,4,8,16]. Although both sexes displayed non-random associations, they also had sex-specific association patterns. Males generally formed stronger bonds than other members of the population, as indicated by a higher average HWI for male–male associations than female–female or male–female associations. Some males formed strong pair bonds, which were the basis for two connected groups, while two males were only loosely associated with the others. Only females had a proportion of non-zero elements smaller than random, indicating intentional avoidance of other individuals of the same sex. As a result, Golfo Dulce bottlenose dolphins had a society with loose female associations and strong bonds between males.

Contrary to our predictions, the type of society we observed in this study was not unique, rather it fit the pattern described for the genus in other regions of the world [3–8]. Strong bonds in males were present, yet given the relatively short duration of the study, they may not represent stable partnerships or male alliances, such as those seen in Shark Bay [3,6,10]. Long-term data are needed to determine the nature of the male bonds in Golfo Dulce.

Despite the overall similarity to other populations, we also detected key differences in Golfo Dulce. Unlike bottlenose dolphins in the Bahamas, which had a few male pairs with very high associations and a majority of males with lower associations [4], the Golfo Dulce population had a majority of males with high associations and only two males with low associations. The two males with low associations bear similarity to the unpaired males observed in Sarasota Bay. However, unlike dolphins in Sarasota Bay [8,15], all of the males which fitted Owen *et al*.'s [15] definition of partnered males in Golfo Dulce had one or two additional males with which they also strongly associated. In this regard, our results more closely resemble the ‘odd-male-out’ dynamics described in Shark Bay [3]. Finally, Golfo Dulce bottlenose dolphins also did not display as many strong associations between males as those in Doubtful Sound [16]. Thus, the pattern observed indicates a social structure with more similarities to both Shark Bay and Sarasota Bay, rather than similarities with Doubtful Sound due to shared fjord-like topography. Our results indicate that, despite the uniqueness of Golfo Dulce, differences in social structure relative to other populations were a matter of degree and suggest that phylogenetic constraints may also be important in understanding variations in the social structure of dolphins [18]. They also emphasize the uniqueness of the social structure of bottlenose dolphins in Doubtful Sound.

Strong male–male bonds often facilitate cooperation for female acquisition and defence [6]. Although further research is required to examine this question in Golfo Dulce, reproductive strategies may also be the basis for the strong bonds observed among males. The amount of scarring observed in males relative to females is suggestive of greater male aggression and male–male competition. If the competition-for-females hypothesis is correct, it is expected that females with older calves will show the scars they obtained during their relatively recent receptive period, whereas females with younger calves have probably had sufficient time since her last receptive period for the scars to fade due to quick repigmentation of damaged tissue [52]. While we did not find significant differences in scarring between receptive and non-receptive females, it is interesting that they did not follow the pattern predicted by the competition-for-females hypothesis, a finding which contrasts with previous research in Shark Bay, Australia [11], and that warrants future study in Golfo Dulce. Although we cannot be certain of the circumstances in which the scars were obtained, we speculate that they were produced by adult males; however, we also do not exclude the possibility that females produced them.

The random male–female associations we observed suggest that selective mating was probably not occurring given that associations between males and females are linked with mating opportunities [16]. This conclusion is made from previous observations that mating promotes mixed-sex groups [54], 76% of the interaction time of mixed-sex groups involves coercive behaviour [12], males preferentially associate with receptive females [15], male–female associations are correlated with reproductive seasonality [8] and associations between females and most male subgroups are higher and more consistent while females are receptive [3]. The pattern of observations just described is unsurprising as females are a primary resource for the reproductive success of males [10]. Unfortunately, we have no evidence of the proportion of male–female associations in this population that were sexual or reproductive in nature. Further research is required to disentangle the nature of the random male–female associations among Golfo Dulce bottlenose dolphins.

### 4.2. Feeding and non-feeding social structure

Overall, there were many similarities in the social structure of bottlenose dolphins between feeding and non-feeding behaviours. There was a high correlation in HWI between the two behavioural states. In addition, average group size, standard deviation of HWI, non-zero elements and community division were similar for both behaviours. The patterns we detected can give us information about what processes, as indicated by behavioural state, may be driving the overall social structure of Golfo Dulce bottlenose dolphins. Specifically, the high lagged association rates between males during non-feeding suggest the importance of strong, long-term bonds when they were not engaged in feeding. Perhaps, these strong bonds are established during non-feeding for acquisition of mates, as observed in some regions where males form such bonds for female coercion and defence as well as for male–male competition over a mosaic of overlapping ranges [6,10,11,18]. Hence, individuals must depend on social networks rather than community defence or mating-season defence [55]. The higher sLAR for male–female associations during non-feeding further supports this hypothesis because an increase in intersexual associations at those times might be related to mating.

Although we had anticipated male–male associations during feeding to be similar to associations during non-feeding, this was not the case. The steep decline in association between males during feeding activities suggests that there was less pressure towards pair fidelity at those times, rather than consistently temporally stable associations due to mating system constraints. This result indicates either solitary foraging (which we sometimes observed) or that foraging success did not depend on cooperation or with whom an individual male cooperated and that non-foraging associations between males were not harmed by social fluidity during feeding. Additional support for this hypothesis is provided by the mean HWI values of the population and of males, which were higher than random during non-feeding but not during feeding behaviours. Thus, two pieces of evidence support the conclusion that strong associations by males were unrelated to food acquisition and the hypothesis that they were driven by mate acquisition. Additionally, while not anticipated, the presence of increased social fluidity during feeding rather than maintaining restrictions on association partners may increase the behavioural resilience of this population in the face of losses.

Surprisingly, the social structure of females was more similar between behavioural states than the one of males: high connectivity and low but stable lagged association rates in both behavioural states. This finding coupled with circumstantial evidence of male aggression based on a larger amount of scarring in males than females, suggests that females were using many loose connections for defence from males in both behavioural states rather than associating flexibly to maximize foraging. In comparison with previous findings on female associations, in both Shark Bay and the Bahamas females display fewer strong associations than males and many low-level associations [3,4]. In Shark Bay, these association patterns resulted in a network composed of long chains of associates [3] and a high percentage of associations which were not preferred in any behavioural state [25]. In the Bahamas, it was also noted that high female associations were related to reproductive status [4], which has also been predicted to shape formation of loose female bonds [9]. Alternatively, Möller [18] predicts moderate female social bonds to form primarily based on kin and incorporate shorter-term non-kin associations, rather than reproductive status. In either case, formation and maintenance of social bonds aids in defence from predators [9,18,25] and male harassment when sexual conflict is present in the population [18]. These benefits would have been present during both feeding and non-feeding behaviours, as both displayed high levels of connectedness, both within and between clusters. However, slightly greater connections between clusters during feeding may have provided an additional benefit in the transmission of food information, as predicted from our hypotheses, or have been a by-product of utilizing the same food sources.

Sex-specific patterns and their putative causes were supported by findings of many similarities in the overall social structure of bottlenose dolphins between feeding and non-feeding behaviours. There was a high correlation in HWI between the two behavioural states. In addition, average group size, standard deviation of HWI, non-zero elements and community division were similar for both behaviours. The similarities between feeding and non-feeding behaviours that we observed support the suggestion made by Gero *et al*. [25] that constraints in social structure are based on mating strategies, as the structure observed facilitates behavioural patterns that are advantageous for mating. For example, strong male bonds facilitate male alliances [6], which engage in female coercion [6,11]. Additionally, the formation of large groups of loosely bonded females facilitates defence against male harassment [12] or calf predation [56]. Female associations may also facilitate reproductive success [57] and shape their calves' social development [56,58,59].

## 5. Conclusion

Although this study gathered data over 16 months as opposed to multiple years, we were able to describe the social structure of bottlenose dolphins from a unique environment near the equator. Long-term studies in Golfo Dulce, similar to those carried out in other places [4,6,8] should examine the effects of deaths, births, immigrations and emigrations on the social structure of bottlenose dolphins as well as any inter-annual variations that may occur [4]. Given that the discovery rate of new individual dolphins reached a plateau relatively fast, we are confident that most of the identifiable population was photographically captured during the study. We were conservative in sexing individual dolphins by requiring visible genitals or close association with a calf through every single sighting. However, further studies will enhance knowledge of the social structure that we describe by sexing all members of the population.

Our study indicates that bottlenose dolphins from a unique environment close to the equator have a similar social structure as that of populations from other latitudes. This population clearly displays strong bonds between males which extend beyond pairs, though we cannot yet conclude whether first-order or multilevel alliances are present. It thus provides further evidence that bottlenose dolphin society is very similar throughout the world with minor variations. It also suggests that the social structure of bottlenose dolphins in Doubtful Sound [16] is indeed unique among the genus *Tursiops*. We also provide evidence that leads us to posit the hypothesis that the strength of male bonds of Golfo Dulce bottlenose dolphins was driven by male competition for access to females rather than by the influence of food resources, as described elsewhere [9,10,25,27,60], and that female associations during feeding are constrained by mating strategies. This research also demonstrates a difference in population-wide social connections between behavioural states, particularly in temporal patterns for males. Under this scenario, male–male competition for access to females was strongest during non-feeding behaviour and most relaxed during feeding. This previously unreported finding highlights the importance of considering behavioural state when examining individual bonds and the behavioural plasticity for which the bottlenose dolphin is well known [16,61–66].

## Supplementary Material

Golfo Dulce Tursiops surveys (in GolfoDulceRSOS.xls) contains data from each Tursiops truncatus sighting during our surveys conducted in Golfo Dulce from September 1991 to December 1992, as well as age, sex, and calf information for each identified individual.

## Supplementary Material

Scarring data (in Scars data to RSOS.xls) contains the data recorded from photographs of fin scarring. Information on each individual, as well as the pixels of their total fin and different scar types on their fin are included.

## Data accessibility

Data are included in the article's electronic supplementary material.
